# Mapping metabolite change in the mouse brain after esketamine injection by ambient mass spectrometry imaging and metabolomics

**DOI:** 10.3389/fpsyt.2023.1109344

**Published:** 2023-05-10

**Authors:** Guan-Xi Liu, Ze-Lin Li, Su-Yan Lin, Qian Wang, Zheng-Yi Luo, Kai Wu, Yan-Lin Zhou, Yu-Ping Ning

**Affiliations:** ^1^The First School of Clinical Medicine, Southern Medical University, Guangzhou, China; ^2^The Affiliated Brain Hospital of Guangzhou Medical University, Guangzhou Huiai Hospital, Guangzhou, China; ^3^The Second Affiliated Hospital of Guangzhou Medical University, Guangzhou, China; ^4^School of Biomedical Sciences and Engineering, South China University of Technology, Guangzhou International Campus, Guangzhou, China

**Keywords:** ketamine, depression, antidepressant, spatial metabolomics, mass spectrometry imaging

## Abstract

Ketamine is a new, fast, and effective antidepression treatment method; however, the possible dissociation effects, sensory changes, abuse risk, and the inability to accurately identify whether patients have a significant response to ketamine limit its clinical use. Further exploration of the antidepressant mechanisms of ketamine will contribute to its safe and practical application. Metabolites, the products of upstream gene expression and protein regulatory networks, play an essential role in various physiological and pathophysiological processes. In traditional metabonomics it is difficult to achieve the spatial localization of metabolites, which limits the further analysis of brain metabonomics by researchers. Here, we used a metabolic network mapping method called ambient air flow-assisted desorption electrospray ionization (AFADESI)-mass spectrometry imaging (MSI). We found the main changes in glycerophospholipid metabolism around the brain and sphingolipid metabolism changed mainly in the globus pallidus, which showed the most significant metabolite change after esketamine injection. The spatial distribution of metabolic changes was evaluated in the whole brain, and the potential mechanism of esketamine’s antidepressant effect was explored in this research.

## 1. Introduction

Ketamine is a non-competitive N-methyl-D-aspartate (NMDA) glutamate receptor antagonist. It has been used in clinics for decades as an anesthetic. In recent years, more and more studies have shown that ketamine has an immediate and effective antidepressant effect and can significantly reduce the suicidal tendency of patients with refractory depression. Ketamine can quickly alleviate depressive symptoms and can last for 7 days. The short-term repeated application and even the remission period can reach more than 18 days (1–3). Ketamine has two enantiomers: arketamine and esketamine, of which the latter shows a five-times higher affinity for the NMDA receptor than arketamine (4). The FDA approved esketamine for treatment-resistant (TR) major depressive disorder (MDD) in 2019 and MDD accompanying suicidal ideation in 2020 (5). However, the possible dissociation effects, sensory changes, abuse risk, and the inability to accurately identify whether patients have a significant response to ketamine limit its clinical use (6). Therefore, further exploration of the antidepressant mechanisms of ketamine can further optimize the application of ketamine in antidepressant treatment and develop a safer, faster, and more effective treatment for depression.

Metabolites are the products of upstream gene expression and protein regulatory networks and play an essential role in various physiological and pathophysiological processes (7, 8). In recent years, metabolomics technology has developed rapidly. Due to the crucial physiological role of metabolites, these metabonomic techniques have been widely used to identify biochemical disorders in diseases (9). Recently, some researchers have focused on the metabolite changes in some brain regions after sub-anesthetic ketamine injection based on liquid chromatography-mass spectrometry (LC-MS), which has given us some hints to a certain extent (10–12). However, due to the heterogeneity of brain tissue and the extensive interrelationship between various subregions, in traditional metabonomics, it is difficult to achieve the spatial localization of metabolites, which limits the further analysis of brain metabonomics by researchers. Mass spectrometry imaging (MSI) technology has become a promising imaging system because of its high flux, convenience, time-saving and labor-saving qualities, and that it has no need for specific chemical markers. It can map the quantitative and spatial distribution of proteins, metabolites, and lipids in the brain with high sensitivity and high throughput (13–15). At present, more and more studies have been conducted to evaluate the changes of metabolites in various disease models using mass spectrometry imaging. Sighinolfi et al. used a non-targeted mass spectrometry MALDI imaging approach and studied the changes and distribution characteristics of various lipids in different brain regions of rats after high-fat diet (16). Another study used the MSI to detect the cortical lipid changes from preclinical to severe stages of Alzheimer’s disease (17). However, research about the metabolic changes induced by ketamine injection based on mass spectrometry imaging is still lacking.

Here, we used a metabolic network mapping method called ambient air flow-assisted desorption electrospray ionization (AFADESI)-MSI (18), which has shown a powerful ability to identify nearly all kinds of important small polar molecules. Researchers can use this technology to detect broad-spectrum metabolites and sensitively detect the changes before and after drug interventions (19). We not only detected the metabolic alterations after ketamine injection but also depicted the metabolic networks at the spatial level, which clearly and comprehensively evaluated the metabolic pathway changes of the whole brain after ketamine injection.

Our research found that esketamine could mainly regulate glycerophospholipid metabolism and downregulated phosphatidylcholine (PC) and phosphorylethanolamine (PE) at the whole brain level, and that glycerophospholipid metabolism changed in almost every brain subregion. We found that the pallidal had the most differential metabolites detected, and sphingolipid metabolism mainly and significantly changed in the pallidal. The ventral pallidum showed the most significant changed metabolites in subregions of pallidal; we found substantial changes in these membrane lipids and observed other changed metabolites that could interact with genes that contribute to the depressive state. Regarding the importance of the ventral pallidum on the reward system, we infer that the ventral pallidum could be the main target of esketamine’s antidepressant effects and is possible through the regulation of metabolites, especially membrane lipids.

## 2. Materials and methods

### 2.1. Animals

Twenty Adult male C57/BL6 mice (Guangdong Medical Laboratory Animal Center, Guangzhou, China) at the ages of 8 weeks were used in the present study. The mice were housed five per cage under a 12 h light/dark cycle (lights on from 7:00 a.m. to 7:00 p.m.) at room temperature (22°C–26°C). The mice had free access to water and food. Animals were randomly assigned to the treatment groups. After the forced swim test, we randomly allocated four mice to each group to do the (AFADESI)-MSI. The behavioral test was done at 10:00 a.m. All procedures involving animals were approved by the Animal Experimentation Ethics Committee of Guangzhou Medical University and were conducted in accordance with guidelines of the National Institutes of Health on the care and ethical treatment of animals.

### 2.2. Reagent

Frozen slice embedding agent (Leica, Germany), Eosin Y-solution 0.5% aqueous (Sigma, United States), Hematoxylin, Mayer’s (Sigma, United States); Dako Bluing Buffer (Qizhong Information Co., Ltd., Shanghai), acetonitrile (MS grade, Thermo Fisher, United States), formic acid (HPLC grade, Millipore, Germany), and Watsons Water (Watsons, HongKong, China).

### 2.3. Ketamine treatment

Esketamine hydrochloride (Jiangsu HengRui Pharmaceutical Co., Ltd., Jiangsu, Fujian, China) was dissolved in saline for the injections. Mice were intraperitoneally (i.p.) injected with vehicle or esketamine (10 mg/kg) 1 h before forced swim tests. The dosage of ketamine was based on peer studies and our previous pre-experiment (20).

### 2.4. Forced swim test

Forced swim tests (FSTs) were used to detect the antidepressant effects of esketamine.

Animals were individually placed in a cylinder, 20 cm in diameter with water up to a height of 20 cm (23°C ± 0.5°C), and swam for 6 min under normal light. Water depth was set to prevent animals from touching the bottom with their tails or hind limbs. Animal behavior was videotaped from the side. The immobile time during the last 4 min of the test was counted offline by an observer blinded to animal treatment. Immobile time was defined as the time when animals remained floating or motionless with the exception of movements necessary for keeping balance in the water.

### 2.5. Tissue collection and sectioning

At 23 h after the forced swim test, mice were firstly anesthetized with isoflurane (Ruiwode Life Technology Co., Ltd., Shenzhen, China) with 2.5% concentration to induce anesthesia and 1.5% concentration for anesthesia maintenance. After they were deeply anesthetized, we quickly cut off the head of the mouse with straight scissors and put it on ice, cutting the scalp to expose the skull. Then, the skull was carefully dissected with bone biting forceps and the whole brain encompassing the regions from the olfactory bulb to the hindbrain structure from each animal was quickly removed with a crowbar and placed into liquid nitrogen, after which it was transferred to −80°C before being sectioned. Eight mouse brains used for the research were cut into sagittal sections at 10um. Leica CM1950 frozen slicer (Leica, Germany) was used to make the slices, guided by the rat brain in stereotaxic coordinates (21). Sections were stored at −80°C before further analysis. They were desiccated at −20°C for 1 h and then at room temperature for 2 h before MSI analysis. Meanwhile, slices adjacent to the selected slice were used for hematoxylin–eosin (H&E) staining.

### 2.6. Data acquisition

The analyses were carried out with an AFADESI23-MSI platform in tandem with a Q-Orbitrap mass spectrometer (Thermo Fisher, United States). In positive ion scanning mode, the spray solvent was composed of acetonitrile: water = 80: 20 (V/V, containing 0.1% formic acid); acetonitrile: water = 80: 20 (V/V) was used for the negative mode. The solvent flow rate was 5 μL/min. Mobile platform parameters and mass spectrum scanning parameters were as follows:

Mobile platform parameters:

**Table:** 

Parameters	Value
X-axis scanning speed (Vx, mm/s)	0.4
Y-axis step spacing (Dy, mm)	0.1
Delay time per line (Dt, s)	7
Resolution ratio (μm*μm)	100*100

Spectrum scanning parameters:

**Table:** 

Parameters	Value
Spray voltage	±0 V
Tube Voltage	±0 V
Sheath gas flow rate	0
Aux gas flow rate	0
Sweep gas flow rate	0
Capillary temp	350°C
Aux gas heater temp	0
Scan mode	Full MS
Scan range	70–1,000 Da
Resolution	60,000
Spray gas press	0.6 MPa
Spray angle	60°
Distance from sprayer to surface	0.7 mm
Distance from spray to guide tube	3 mm
Distance from orifice to guide tube	10 mm
Extracting gas	45 l/min

### 2.7. Data processing and bioinformatics analysis

The raw data was converted to ImzML file format by imzMLConverter processing software, and imported into the Cardinal software package for background deduction, peak alignment, and peak filtering. Peaks were matched based on proximity of their m/z-values, according to tolerance, in 5 parts-per-million (ppm). The experiment contained a known reference peak with a constant abundance throughout the dataset, using a reference to normalize all spectra data. The proportions of pixels where a peak was detected at each m/z-value were calculated, and only peaks with frequencies greater than 0.01 were retained. The SmetDB database and pySM annotation framework were used to annotate metabolites in high-resolution mass spectrometry imaging. The metabolites which had spatial chaos > 0.9, a spectral isotope measure > 0.1, and a spatial isotope measure > 0.1 were selected for the next step in the analysis. Metaboanalyst 5.0 was used to conduct the pathway analysis based on the KEGG (Kyoto Encyclopedia of Genes and Genomes) and to draw the Metabolite-Gene-Disease Interaction Network, the disease association based on the Human Metabolome Database (HMDB) from the Human Metabolome Project’s literature curation team. The chemical and human gene associations were extracted from STITCH, such that only highly confident interactions were used; most associations in STITCH are based on co-mentions highlighted in PubMed abstracts, including reactions from similar chemical structures and similar molecular activities.

### 2.8. Statistical analysis

SPSS 25.0 was used to analyze the data, which were shown as mean ± SEM. *t*-tests (*n* = 4 mice each group, one slice from each mouse) were used to make the comparison between groups. The criterion for statistical significance was *p* < 0.05, shown as *, and *p* < 0.01, shown as **. The sample size was referenced from similar peer research (16, 17, 19).

## 3. Results

### 3.1. Metabolic change in whole brain level

**Figure 1:**
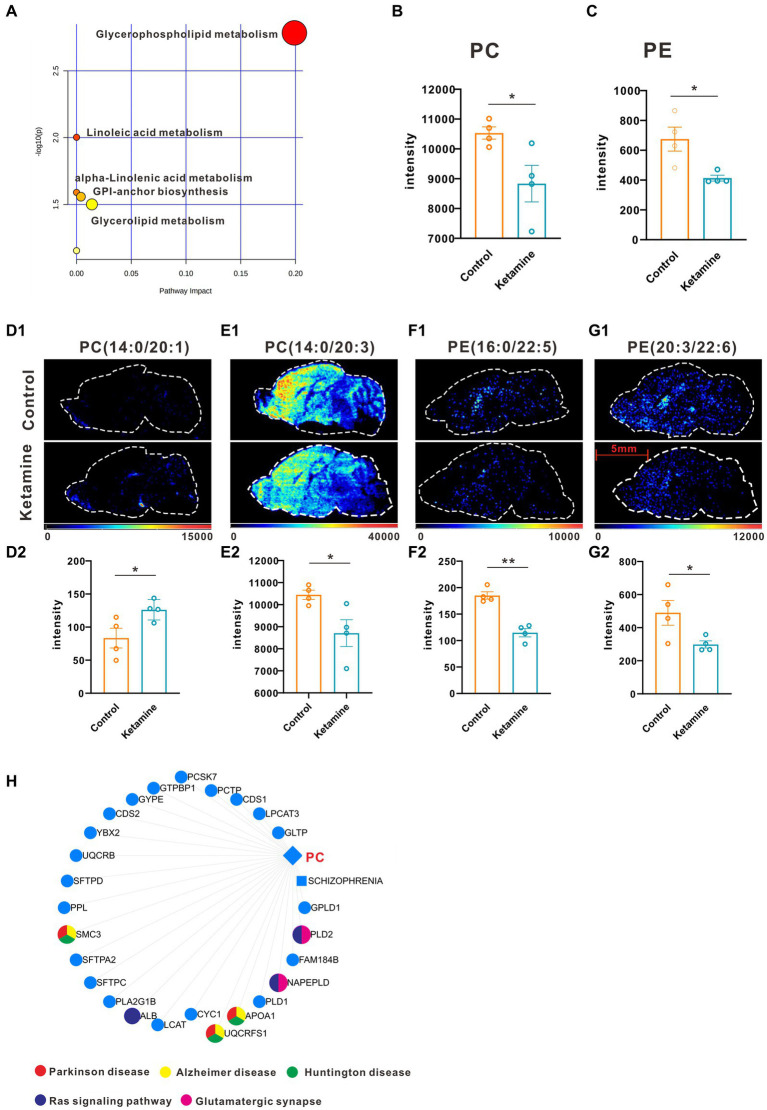
One slice from each brain, four mouse brains for each group. **(A)** Pathway analysis using Metaboanalyst 5.0 based on KEGG. **(B)** The intensity changes of PC, *p* = 0.039, a two-tail independent *t-*test was used for statistical analysis, t = 2.632, df = 6, *p* = 0.039. **(C)** The intensity changes of PE, a two-tail independent *t*-test was used for statistical analysis, t = 3.161, df = 6, *p* = 0.0196. **(D1–G1)** The spatial distribution of changed metabolites belonging to glycerophospholipid metabolism. **(D2–G2)** The intensity changes of each metabolite, a two-tail independent t-test was used for statistical analysis, D2:t = 2.547, df = 6, *p* = 0.044; E2:t = 2.711, df = 6, *p* = 0.0351; F2:t = 6.73, df = 6, *p* = 0.0005; G2:t = 2.459, df = 6, *p* = 0.0492. **(H)** Metabolite-Gene-Disease Interaction Network based on Metaboanalyst 5.0. The different colors represents the different pathways; the points have not been marked, which indicates that it has not been enriched in pathways closely related to neuro-mental disorders. Statistical significance is *p < 0.05, **p < 0.01.

Firstly, we conducted the FST to evaluate the antidepressant effect of esketamine, and the results showed the esketamine injection could significantly reduce immobility duration (Supplementary Figures S1A,B). After the brain slices were prepared, mouse brain sections were scanned by electrospray ionization (ESI) probe pixel by pixel. The desorbed ions were transported to and analyzed by a high-resolution mass analyzer both in positive and negative ion modes. The H&E staining of the sagittal sections (Supplementary Figure S1C) showed the selected sections used for analysis, and we chose those sections in order to cover as much as possible of the brain subregions which researchers have mainly focused on, such as the medial prefrontal cortex, hippocampus, nucleus accumbens, and ventral tegmental area (22–24). By using (AFADESI)-MSI metabolomics to detect brain slices from the ketamine-treatment and control group mice, a total of 702 characteristic peaks were detected in negative mode and 318 in positive mode and were used for subsequent differential expression analysis. We found 11 differential metabolites between the ketamine-treatment group and the control group by the criteria of fold change > 1.2 and *p* < 0.05 (Table 1). Then, we ran the pathway enrichment analysis based on the KEGG; the data showed that the pathways enriched were almost concentrated in fatty acid metabolism. Glycerophospholipid metabolism had the highest impact score (Figure 1A), which may prompt fatty acid metabolism. In particular, glycerophospholipid metabolism may contribute to the action mechanisms of sub-anesthetic ketamine, according to some previous studies which have already reported its relationship with depression (25, 26). Figures 1B,C show the changed metabolites belonging to glycerophospholipid metabolism, and the different subtypes of PC and PE are shown in Figures 1D–G. The Metabolite-Gene-Disease Interaction Network offered two changed metabolites: PC, triglyceride (TG), have to match the interactions with genes or disease. PC—members of glycerophospholipid metabolic pathway, shows the most complicated interaction network. Some genes that interact with PC, were matched with central nervous system diseases pathway ,the Ras signaling pathway, and glutamatergic synapse pathway, which has already been confirmed to contributes to the depression and many other mental disorders (27, 28) (Figure 1H; Supplementary Figure S2). This was a reminder that sub-anesthetic ketamine injection could affect lipid metabolism in the brain, inferring that ketamine may exert antidepressant effects by regulating lipid metabolism, as suggested by previous studies which found there were significant changes in fatty acid metabolism in patients with depression, as well as in animal models (29–31).

**Table 1:** List of differentially expressed metabolites at whole brain level.

m/z	Ion mode	Compound ID	Metabolites	ppm	*p*-value	Fold change	KEGG
782.56	pos	HMDB0007879	PC(14:0/20:1)	4.03	0.04	0.85	C00157
783.56	pos	HMDB0008944	PE(16:0/22:5)	0	0.03	0.84	C00350
847.45	pos	HMDB0013478	PGP(16:0/18:3)	0	0.01	1.31	NA
869.46	pos	HMDB0009939	PIP(16:1)	0	0.05	1.30	C00626
756.55	pos	HMDB0007881	PC(14:0/20:3)	0	0.04	0.83	C00157
792.42	pos	HMDB0012350	PS(14:1/20:4)	0	0.01	1.32	NA
757.55	pos	HMDB0113028	MMPE(35:4)	3.55	0.01	0.80	NA
935.74	pos	HMDB0043067	TG(55:4)	3.41	0.04	1.32	NA
968.77	pos	HMDB0005478	TG(20:4)	1.56	0.03	1.38	C00422
831.56	pos	HMDB0009342	PE(20:3/22:6)	0.49	0.05	0.61	C00350
958.26	pos	HMDB0011604	4,8-Dimethylnonanoyl-CoA	1.11	0.03	1.34	NA

### 3.2. Mapping metabolic network alterations from microregions in the mouse brain

**Figure 2:**
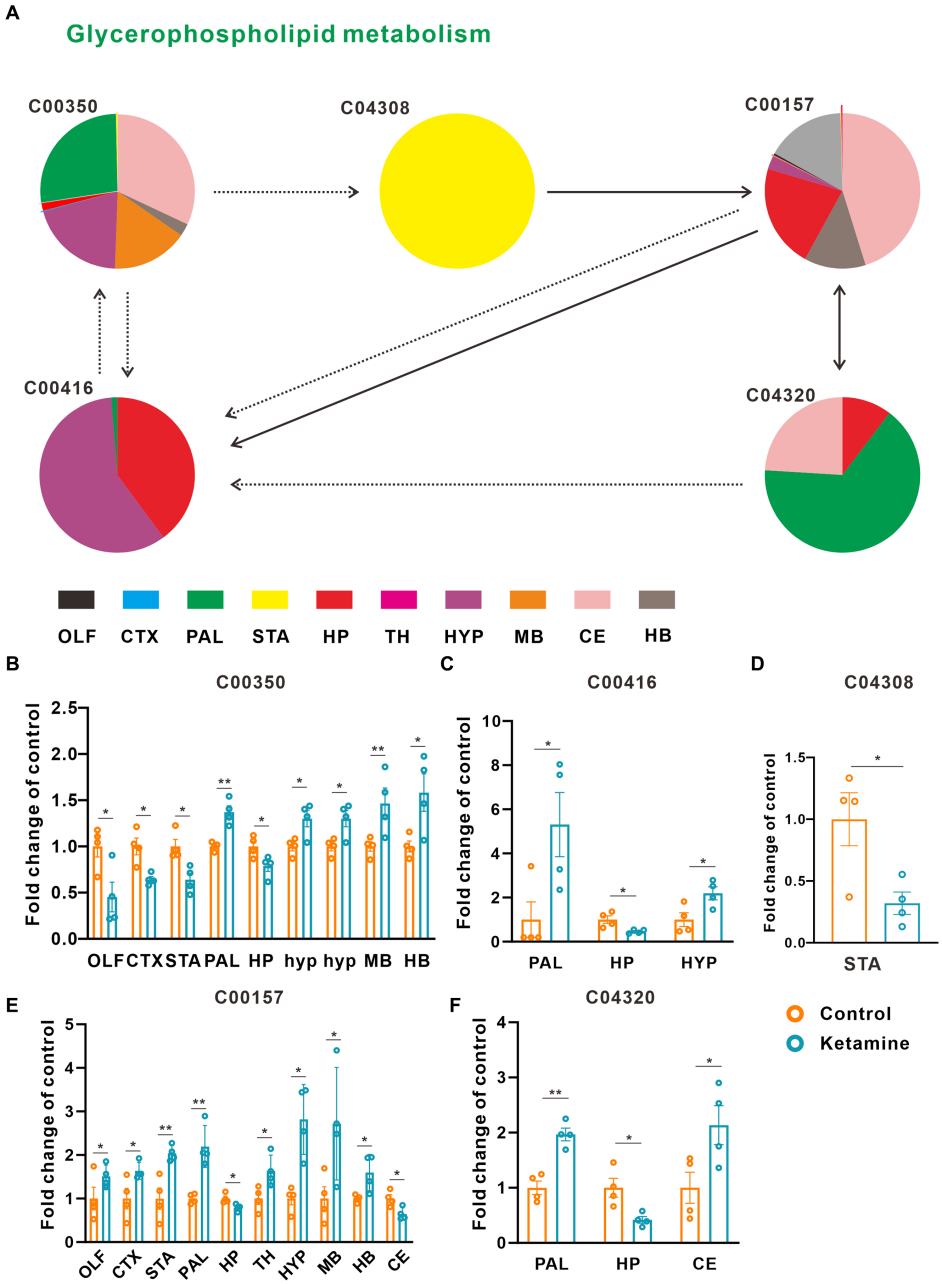
Changed glycerophospholipid metabolism pathways in the ketamine-treatment group brain. **(A)** The sub-region distribution of metabolic changes in the glycerophospholipid metabolism pathways. **(B)** The fold change of C00350 on each subregion, a two-tail independent t-test was used for statistical analysis, OLF: t = 2.782, df = 6, *p* = 0.032; CTX: t = −3.749, df = 6, *p* = 0.01; STA: t = −3.456, df = 6, *p* = 0.014; PAL: t = 5.176 df = 6 *p* = 0.002; HP: t = −2.6, df = 6, *p* = 0.041; MB: t = 2.685, df = 6, *p* = 0.036; HYP: t = 3.005, df = 6, *p* = 0.024; HYP: t = 3.005, df = 6, *p* = 0.034; CE: t = −4.244, df = 6, *p* = 0.005; HB: t = 2.754, df = 6, *p* = 0.033. **(C)** The fold change of C00416 on each subregion, a two-tail independent t-test was used for statistical analysis, PAL: t = 2.589, df = 6, *p* = 0.041; HP: t = −3.067, df = 3.398, *p* = 0.046; HYP: t = 2.77, df = 6, *p* = 0.032. **(D)** The fold change of C04308 on STA, a two-tail independent t-test was used for statistical analysis, t = 2.925, df = 6, *p* = 0.0265. **(E)** The fold change of C00157 on each subregion, a two-tail independent *t*-test was used for statistical analysis, OLF: t = 2.944, df = 6, *p* = 0.026; CTX: t = 2.483, df = 6, *p* = 0.048, STA: t = 3.983, df = 6, *p* = 0.007; PAL: t = 4.844, df = 6, *p* = 0.003; HP: t = −3.110, df = 6, *p* = 0.021; TH: t = 2.801, df = 6, *p* = 0.031; HYP: t = 4.261, df = 3.767, *p* = 0.015;MB: t = 2.452, df = 6, *p* = 0.050; HB: t = 2.807, df = 6, *p* = 0.031; CE: t = −3.127, df = 6, *p* = 0.02. **(F)** The fold change of C04320 on each subregion, a two-tail independent t-test was used for statistical analysis, PAL: t = 5.751, df = 6, *p* = 0.001; HP: t = −3.225, df = 6, *p* = 0.018; CE: t = 2.513, df = 6, *p* = 0.046. One slice from each brain, four mouse brains for each group for analysis. Statistical significance is *p < 0.05, **p < 0.01.

**Figure 3:**
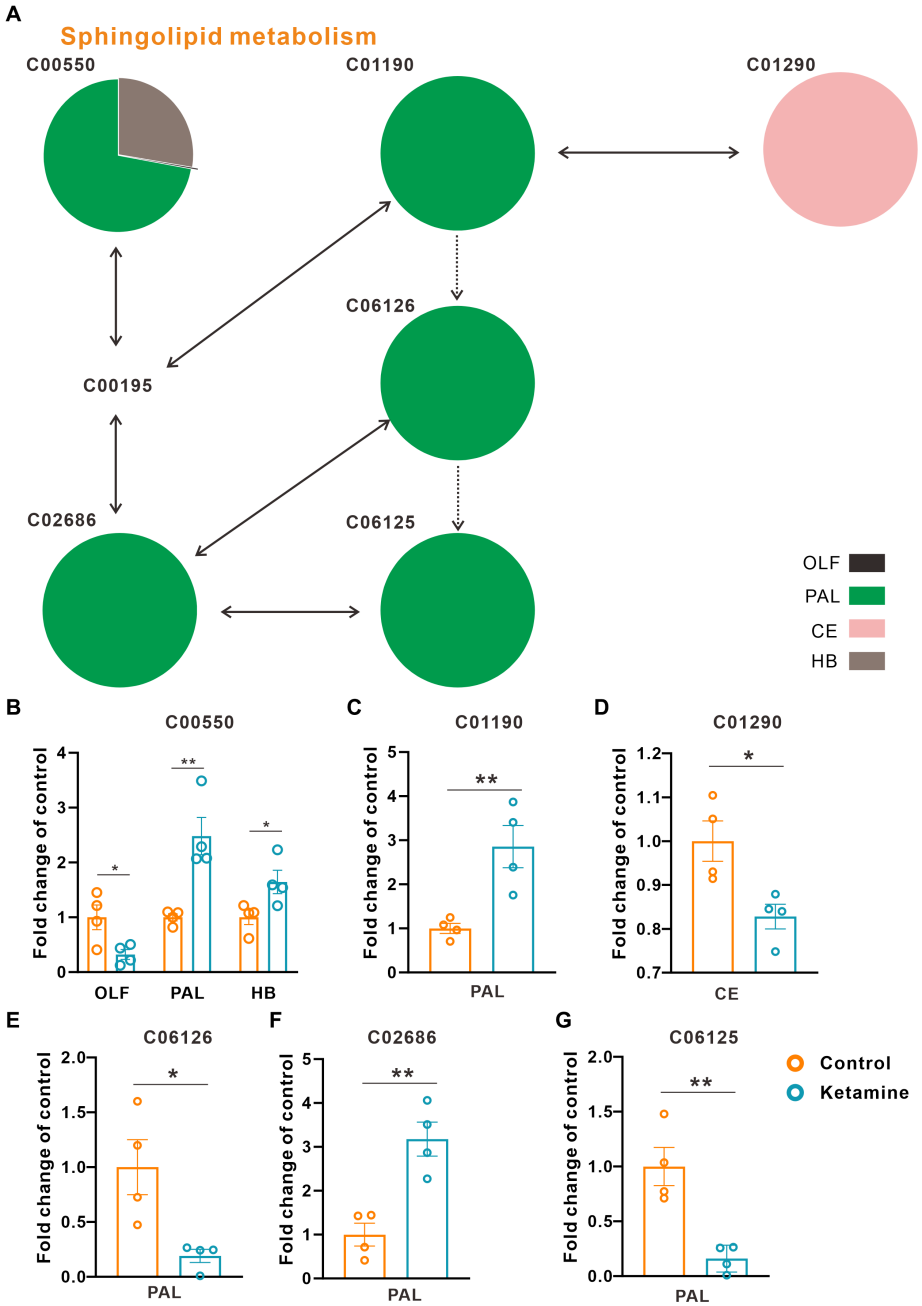
Changed sphingolipid metabolism pathways in the ketamine-treatment group brain. **(A)** The sub-region distribution of metabolic changes in the glycerophospholipid metabolism pathways. **(B)** The fold change of C00550 on each subregion, a two-tail independent t-test was used for statistical analysis, OLF: t = 2.807, df = 6, *p* = 0.031; PAL: t = −2.517, df = 6, *p* = 0.042; HB: t = −4.286, df = 6, *p* = 0.005. **(C)** The fold change of C01190 on PAL, a two-tail independent t-test was used for statistical analysis, t = 3.763, df = 6, *p* = 0.009. **(D)** The fold change of C01290 on CE, a two-tail independent *t*-test was used for statistical analysis, t = 3.183, df = 6, *p* = 0.019. **(E)** The fold change of C06126 on PAL, a two-tail independent t-test was used for statistical analysis, t = 3.141, df = 6, *p* = 0.02. **(F)** The fold change of C02686 on PAL, a two-tail independent t-test was used for statistical analysis, t = 4.668, df = 6, *p* = 0.003. **(G)** The fold change of C06125 on PAL, a two-tail independent t-test was used for statistical analysis, t = 4.532, df = 6, *p* = 0.004. One slice from each brain, four mouse brains for each group for analysis. Statistical significance is *p < 0.05, **p < 0.01.

**Figure 4:**
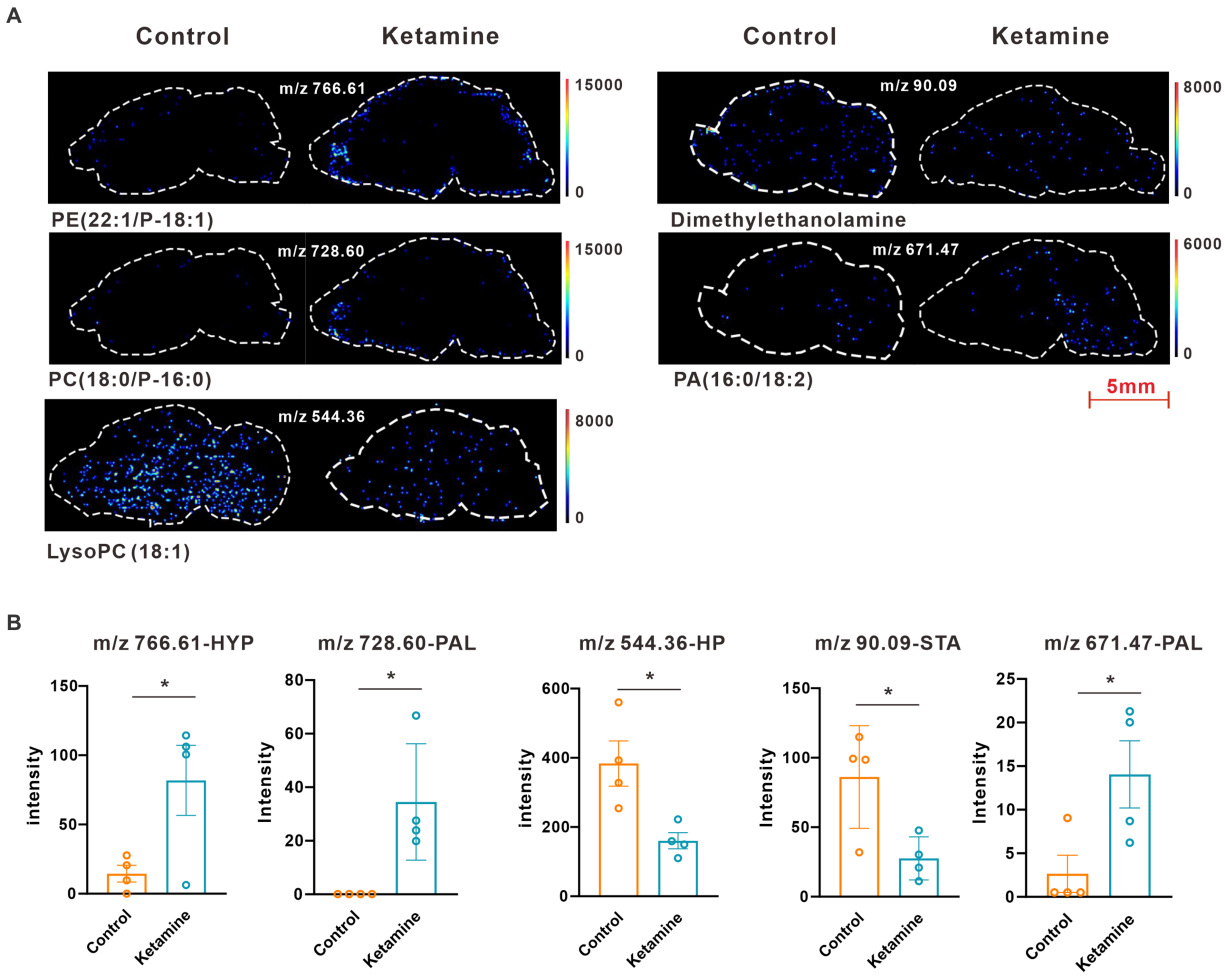
**(A)** Images of AFADESI-MSI showing the most differentially expressed metabolites in brain subregions in the glycerophospholipid metabolism pathways. **(B)** Relative quantitation of metabolites abnormally expressed in brain subregions of the esketamine injection and control groups, a two-tail independent *t*-test was used for statistical analysis, m/z 766.61:t = 2.587, df = 6, *p* = 0.041; m/z 728.60: t = 3.167, df = 6, *p* = 0.019; m/z 544.36: t = 3.183, df = 6, *p* = 0.019; m/z 90.09: t = 2.925, df = 6, *p* = 0.0265; m/z 671.47:t = 2.589, df = 6, *p* = 0.0412. One slice from each brain, four mouse brains for each group for analysis. Statistical significance is *p < 0.05.

**Figure 5:**
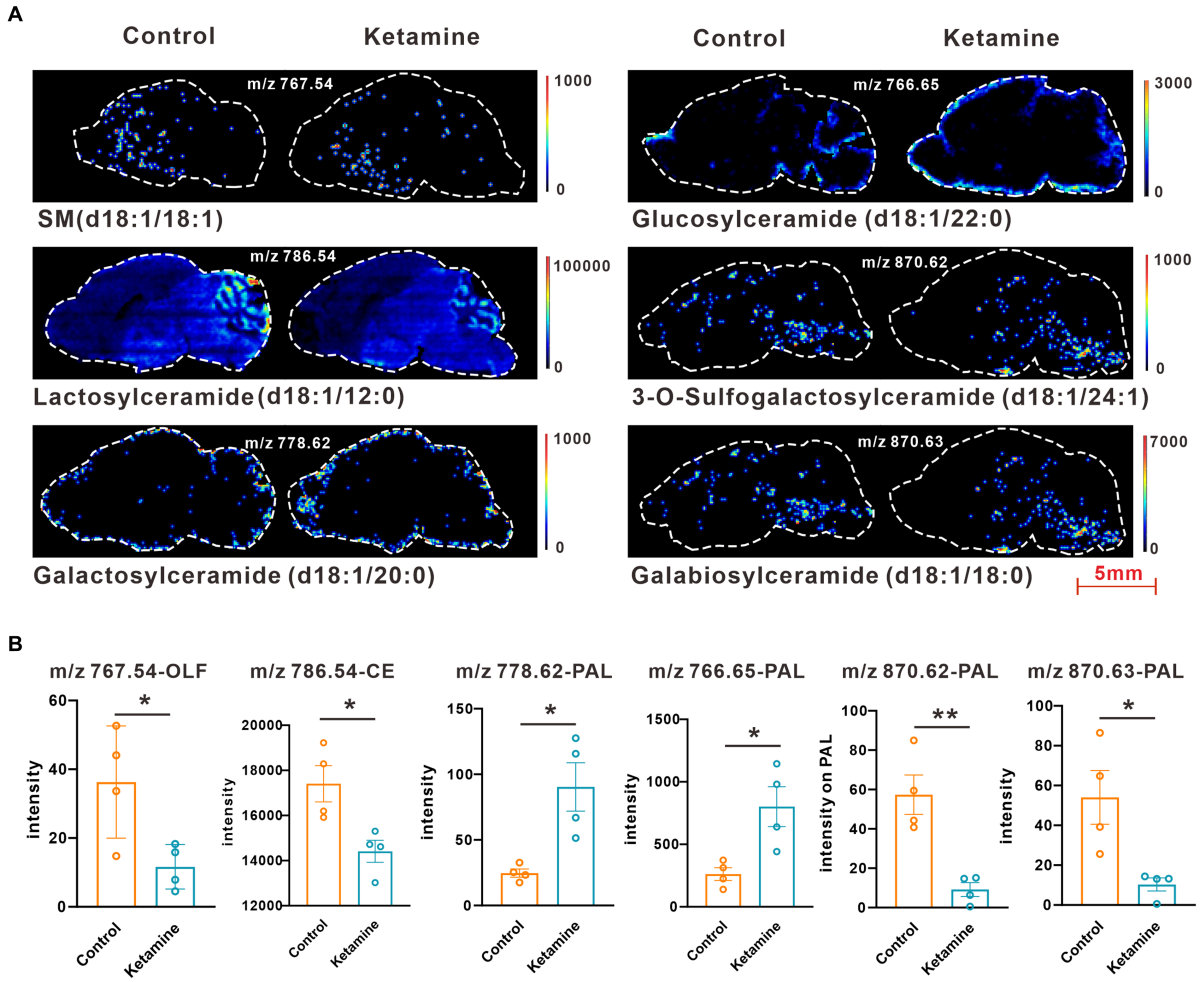
**(A)** Images of AFADESI-MSI showing the most differentially expressed metabolites in brain subregions in the sphingolipid metabolism pathways. **(B)** Relative quantitation of metabolites abnormally expressed in brain subregions of the esketamine injection and control groups, a two-tail independent *t*-test was used for statistical analysis, m/z 767.54:t = 2.807, df = 6, *p* = 0.03; m/z:786.54:t = 3.183, df = 6, *p* = 0.019; m/z:778.62: t = 3.508, df = 6, *p* = 0.013; m/z 766.65: t = 3.221, df = 6, *p* = 0.018; m/z 870.62:t = 4.532 df = 6, *p* = 0.004; m/z 870.63:t = 3.141 df = 6, *p* = 0.02. One slice from each brain, four mouse brains for each group for analysis. Statistical significance is *p < 0.05, **p < 0.01.

At the whole brain level, in total, only 11 differential expressed metabolites have been found, which is due to the heterogeneity of brain regions and complex metabolic profiles. To further evaluate the effects of ketamine injection on brain regions and metabolic pathways’ spatial distribution, we divided the mouse brain into 10 sub-regions based on the Allen brain atlas (32), including the cerebral cortex (CTX), olfactory areas (OLF), striatum (ST), pallidum (PAL), thalamus (TH), hypothalamus (HYP), hippocampus (HP), midbrain (MD), hindbrain (HB), and cerebellum (CE) for the next analysis (Supplementary Figure S3A). Ion peaks were extracted and aligned from the MSI data of each brain microregion of the control and ketamine-treatment groups, and guided by optical images, annotated, and mapped onto the metabolic networks. The *t*-test was used to detect the changed metabolites, with the screening criteria of *p*-value < 0.05, fold change >1.2, and included in KEGG database. Among these subregions, we detected the most differential metabolites in the pallidal, which suggested that the pallidal may be an important target of esketamine (Supplementary Table S1). The pathway analysis showed that glycerophospholipid metabolism and sphingolipid metabolism are significantly affected by esketamine (Supplementary Figure S3B). Figures 2A and 3A show the distribution of changed metabolites in different subregions matched on these pathways, each circle represents a substance in the pathway, and the fan shaped areas of different colors in the circle represent represents the intensity ratios of different regions in the ketamine-treatment group. The fold change of each metabolite is shown in the Figures 2B and 3B. Focusing on glycerophospholipid metabolism, as shown in Figure 4, we found that PE (22:1/P-18:1) shows the most significant changes in the HYP; PC (18:0/P-16:0) and phosphatidic acid (PA; 16:0/18:2) in the PAL; lysophosphatidylcholines (LysoPC) in the HP; and dimethylethanolamine in the STA. In sphingolipid metabolism, sphingomyelin (SM; d18:1/18:1) shows the most significant change in the OLF, lactosylceramide (d18:1/12:0) in the CE, and galactosylceramide (d18:1/20:0), glucosylceramide (d18:1/22:0), 3-O-sulfogalactosylceramide (d18:1/24:1), and galabiosylceramide (d18:1/18:0) in the PAL (Figure 5). These analyses inferred that esketamine shows an impact on fatty acid metabolism in many subregions, especially glycerophospholipid metabolism. Differently from the whole brain level changes, one metabolite showed different expression level changes in different subregions, and subtypes of specific metabolites also showed different change trends in one region, which means esketamine could play a physiological function through regulating glycerol phospholipid metabolism by different regulatory mechanisms in different brain regions. More essential research should be done to explore the role of fatty acid metabolism in esketamine’s anti-depressive effect.

### 3.3. Metabolic alterations in the pallidal

**Figure 6:**
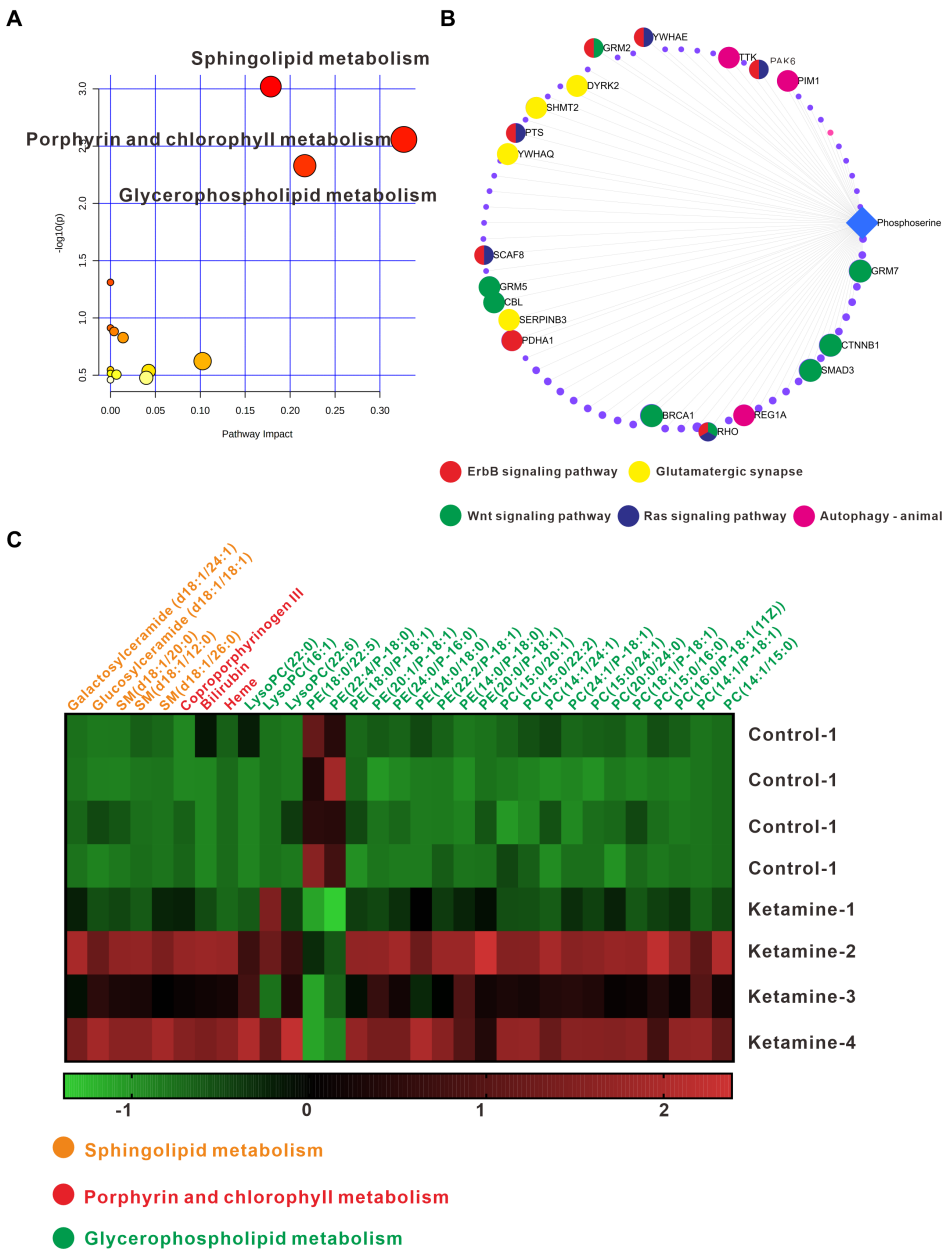
**(A)** Pathway analysis using Metaboanalyst 5.0 based on the KEGG. **(B)** Metabolite-Gene-Disease Interaction Network based on Metaboanalyst 5.0. The different colors represent the different pathways; the points have not been marked, which indicates that it has not been enriched in pathways closely related to neuro-mental disorders. **(C)** Differentially expressed metabolites matching glycerophospholipid metabolism, sphingolipid metabolism, and porphyrin and chlorophyll metabolism; the data underwent Z-Score conversion to more intuitively observe the changes after esketamine injection; the different colors represent the metabolic pathways to which they belong. One slice from each brain, four mouse brains for each group analysis.

In the analysis described above, we found the most differential metabolites in the pallidal, and of the changed metabolites, including in glycerophospholipid metabolism and sphingolipid metabolism, almost all could be detect in pallidal. This was a reminder that the pallidal could be the main target of esketamine, at least on the metabolic level. In addition, peer research has found that the subcortical gray matter volume in the left globus pallidus decreased significantly in MDD patients and PET-CT showed the reduced function of 5-HT(1B) receptors on the ventral parts of the pallidal in humans with MDD (33, 34). Postmortem brain studies also showed that the volume of the lateral globus pallidus was significantly reduced in MDD patients (35, 36). We thus separately analyzed the metabolite changes in the different subregions of the pallidal, including the medial, caudal, and ventral parts (Supplementary Figure S4). Table 2 shows the number of differential metabolites in each subregion; in the medial and ventral parts, 93 and 68 changed metabolites were detected, respectively, with the screening criteria of value of *p* < 0.05 and fold change > 1.2. Only 11 changed metabolites were detected in the caudal part; this suggests that esketamine mainly acts on the medial and ventral parts of the pallidal. The ventral pallidum has been considered the “reward center” in the brain for a long time, and it has been reported that it participates in many psychiatric disorders such as depression, drug addiction, and schizophrenia (37–40). The changed metabolites are summarized in Supplementary Table S2. Pathway analysis showed that the changed metabolites mainly focused on glycerophospholipid metabolism, sphingolipid metabolism, and porphyrin and chlorophyll metabolism (Figure 6A). Figure 6C shows all the metabolite changes matched with the changed pathways. The Metabolite-Gene-Disease Interaction analysis showed that, besides PC, which has been discussed and presented in Figure 1, we also found “phosphoserine” has interacted with plenty of genes that participate in the pathways related to the occurrence and development of depression and many other psychiatric disorders (Figure 6B). This reminds us that esketamine could contribute to fatty acid metabolism and also indirectly affects glutamate synapse function, oxidative stress, and many other signaling pathways to affect the neuronal function of the ventral pallidum to develop antidepressant effects or any side effects. There has not been much research on the role of pallidum, medial region in mental disorders; previous studies have found medial pallidal lesions can affect the performance of rats in a water maze and are associated with anxiety and fear (41, 42). The changed metabolites in the medial pallidal are summarized in Supplementary Table S3. Pathway analysis showed that the changed metabolites mainly focused on glycerophospholipid metabolism, sphingolipid metabolism, and arachidonic acid metabolism (Supplementary Figure S5A). Supplementary Figure S5B shows the changed metabolites belonging to these pathways. In the Metabolite-Gene-Disease Interaction analysis shown in Supplementary Figure S5C, no genes related to neuropsychiatric diseases were found among the interacting genes. The bioinformatics analysis of different subregions of the globus pallidus shows that ventral pallidum could be the main antidepressant action site of esketamine; based on our research and previous peer reports, further essential research for subsequent verification of the role of the ventral pallidum in the antidepressant effect of esketamine has good research value.

**Table 2:** Number of differential metabolites in each subregion of the pallidal.

Subregion	Number of differential metabolites
Ventral pallidum	93
Caudal pallidum	11
Medial pallidum	68

## 4. Discussion

Ketamine, as a new, fast, and effective antidepression treatment method, needs plenty of research to further understand its mechanisms in order for more safe and standardized clinical applications. Metabolites are the products of upstream gene expression and protein regulatory networks and play an essential role in a variety of physiological and pathophysiological processes. Our research focused on the metabolite changes after esketamine injection and used new powerful mass spectrum imaging technology to systematically and comprehensively map the metabolite changes combined with brain regions and metabolic pathways.

At the whole-brain level, we found esketamine mainly affects the glycerophospholipid metabolism pathways, especially PC and PE, corresponding with the previous research reports that animals with stress models show PC and PE have an increasing trend without significance (43). It can be inferred from the analysis results of this study that esketamine may play an antidepressant role by regulating the glycerophospholipid metabolism pathways. Further subregion analysis showed that esketamine could affect glycerophospholipid metabolism in almost every covered subregion, and also sphingolipid metabolism is mainly found in the pallidal. Both glycerophospholipid and sphingolipid are membrane lipids and regulate the membrane’s function as a barrier between the intracellular and extracellular parts. Membrane lipids can regulate synaptic plasticity and also determine the localization and function of proteins within the membrane and activation of these protein. Lipids can influence both exo- and endocytic processes and work within the membrane as second messengers. Lipids can exert physiological function in both directions, relaying signals from the membrane to intracellular compartments as intra-transmitters and also forwarding information to other cells as extracellular transmitters (44, 45). Previous studies into the non-human primate model of depression have detected the upregulation of PC, LysoPC, and PA in the hippocampus of depressive-like monkeys, and our research found the downregulation of these metabolites (26). The sphingolipid system has already been shown to be changed after the stress model in the brain (46) and has been confirmed to be a target of some antidepressant drugs such as paroxetine and desipramine (47). The mechanism of how these lipid acids participate in the pathophysiology of depression and antidepressants is still not precise in the current stage. Whether low-dose ketamine acts on glycerophospholipid and sphingolipid metabolism in the central nervous system has not been reported; only peripheral blood and drug addiction-induced nerve injury models have been reported (48, 49). Further study of the mechanisms of these membrane lipids in depression and exploring how ketamine acts will be of great significance.

Because we detected the most significant metabolite changes in the pallidal, we speculate that ventral pallidum may be an essential target of esketamine. The changes in sphingolipid metabolism mainly occurred in the pallidal part, which indicates that esketamine may regulate pallidal sphingolipid metabolism and then affect related neurons and circuit functions to play an antidepressant role. We further analyzed subregions of the pallidal; the ventral pallidum shows the most changed metabolites and some of which could interact with many genes with participation in pathways that have already been confirmed to regulate depression states.

In conclusion, our research shows that esketamine mainly changed fatty acid metabolism, especially glycerophospholipid and sphingolipid metabolism. The pallidal showed the most significantly changed metabolites, and the ventral pallidum contained plenty of changed metabolites involved in glycerophospholipid and sphingolipid metabolism, and also some other metabolites that could interact with the genes that participate in depression and other mental disorders. This infers that esketamine may exert an antidepressant effect by regulating membrane lipid metabolism, and the ventral pallidum could be the main target of the esketamine-induced metabolism changes.

There are still shortcomings regarding this study. First, this study made a preliminary exploration into the changes of brain metabolic pathways caused by the antidepressant dose of ketamine. In order to find the antidepressant mechanisms of ketamine more accurately, we should further add animal depression models for comprehensive analysis. Second, the sample size of this study was relatively small. In the future, we should combine the depression model on the basis of this discovery, further accurately analyze the metabolic changes caused by ketamine in various brain regions, and conduct basic experimental intervention through the selected targets; this would further enrich the understanding of the antidepressant mechanisms of ketamine, which will help the precise application of ketamine and provide new targets for rapid antidepressants.

## Data availability statement

The raw data supporting the conclusions of this article will be made available by the authors, without undue reservation.

## Ethics statement

The animal study was reviewed and approved by Animal Experimentation Ethics Committee of Guangzhou Medical University.

## Author contributions

G-XL conducted the animal experiments, sample delivery, data analysis, and wrote the manuscript. Z-LL and S-YL helped to make the figures. QW and Z-YL helped to arrange the tables. KW directed the writing of the article. Y-LZ and Y-PN put forward ideas, designed the experiment, and supervised the research process. All authors contributed to the article and approved the submitted version.

## Funding

This work was supported by Guangdong Basic and Applied Basic Research Foundation (grant numbers 2019A1515011366 and 2020A1515011567), Guangdong Basic and Applied Basic Research Foundation Outstanding Youth Project (2021B1515020064), Guangzhou Science and Technology Planning Project of Guangdong (grant number 202103000032), and Science and Technology Plan Project of Guangdong Province (grant number 2019B030316001). The funding source had no role in the study design, analysis, or interpretation of data or in the preparation of the report or decision to publish.

## Conflict of interest

The authors declare that the research was conducted in the absence of any commercial or financial relationships that could be construed as a potential conflict of interest.

## Publisher’s note

All claims expressed in this article are solely those of the authors and do not necessarily represent those of their affiliated organizations, or those of the publisher, the editors and the reviewers. Any product that may be evaluated in this article, or claim that may be made by its manufacturer, is not guaranteed or endorsed by the publisher.
