# Supplementary material for: Mapping metabolite change in the mouse brain after esketamine injection by ambient mass spectrometry imaging and metabolomics
Source: Front Psychiatry. 2023 May 10;14:1109344. doi: 10.3389/fpsyt.2023.1109344 (PMC10206402; doi:10.3389/fpsyt.2023.1109344)
Supplement: Supplementary file 1 [file Data_Sheet_1.pdf]

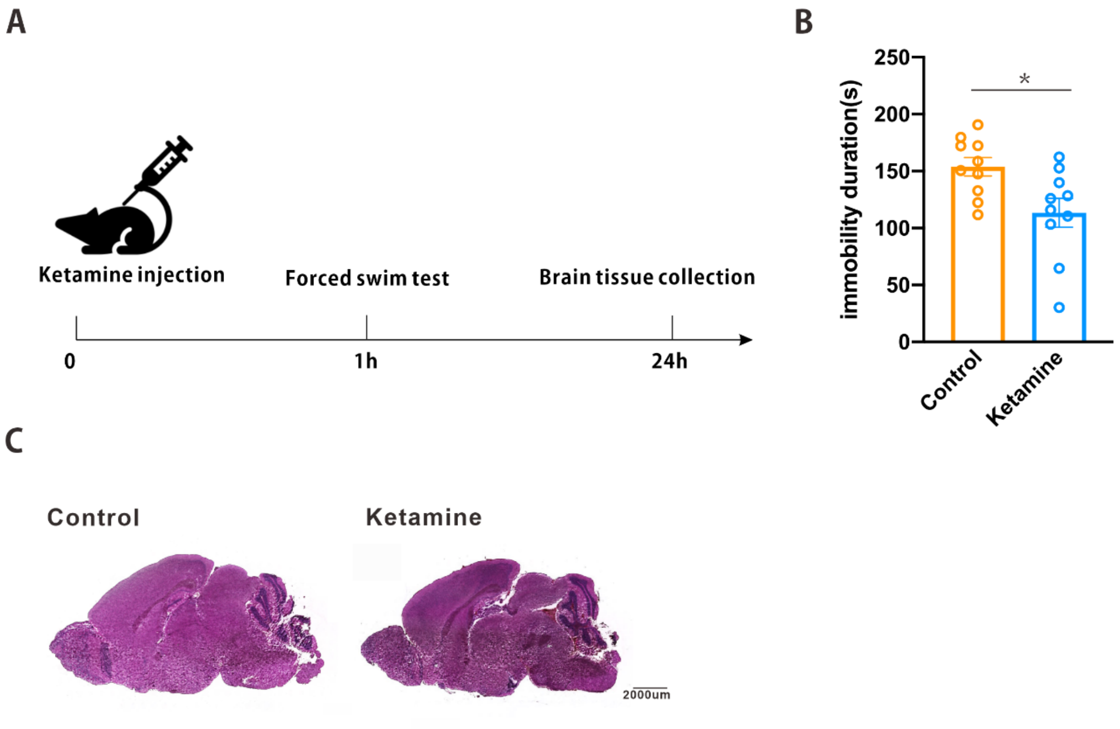

Figure S1. *(A) Schematic Diagram of Animal Experiment Process. (B) s-ketamine reduce the immobility duration in FST. n=10 mice for each group, p=0.015 (C)The H&E staining of the sagittal sections about the selected sections use for analysi*

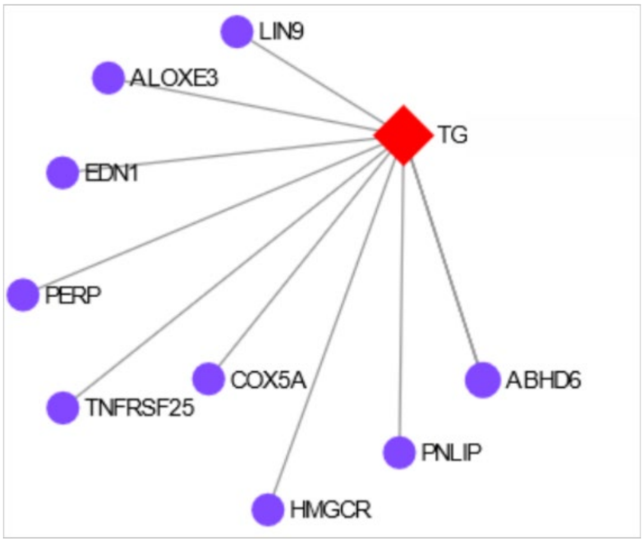

Figure S2. *Metabolite-Gene-Disease Interaction Network for TG based on metaboanalyst5.0.*

A

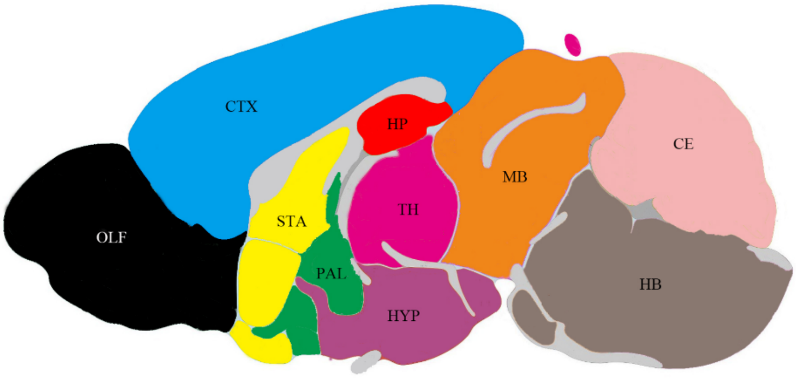

Lateral 0.36-0.48

B

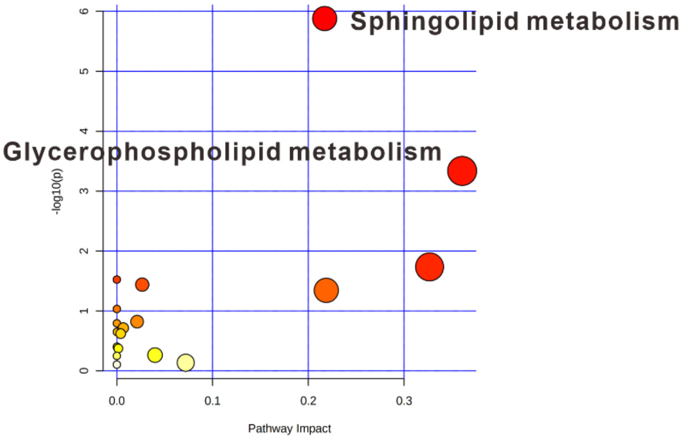

Figure S3. *(A) Schematic Diagram of Subregional Separation (B)Pathway analysis using metaboanalyst5.0 based on KEGG.*

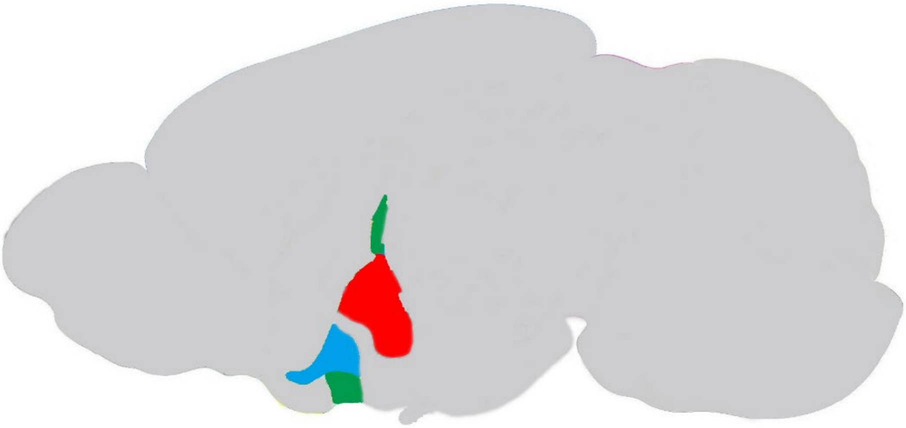

Lateral 0.36-0.48

Figure S4. *Schematic Diagram of Subregional Separation of* pallidal.

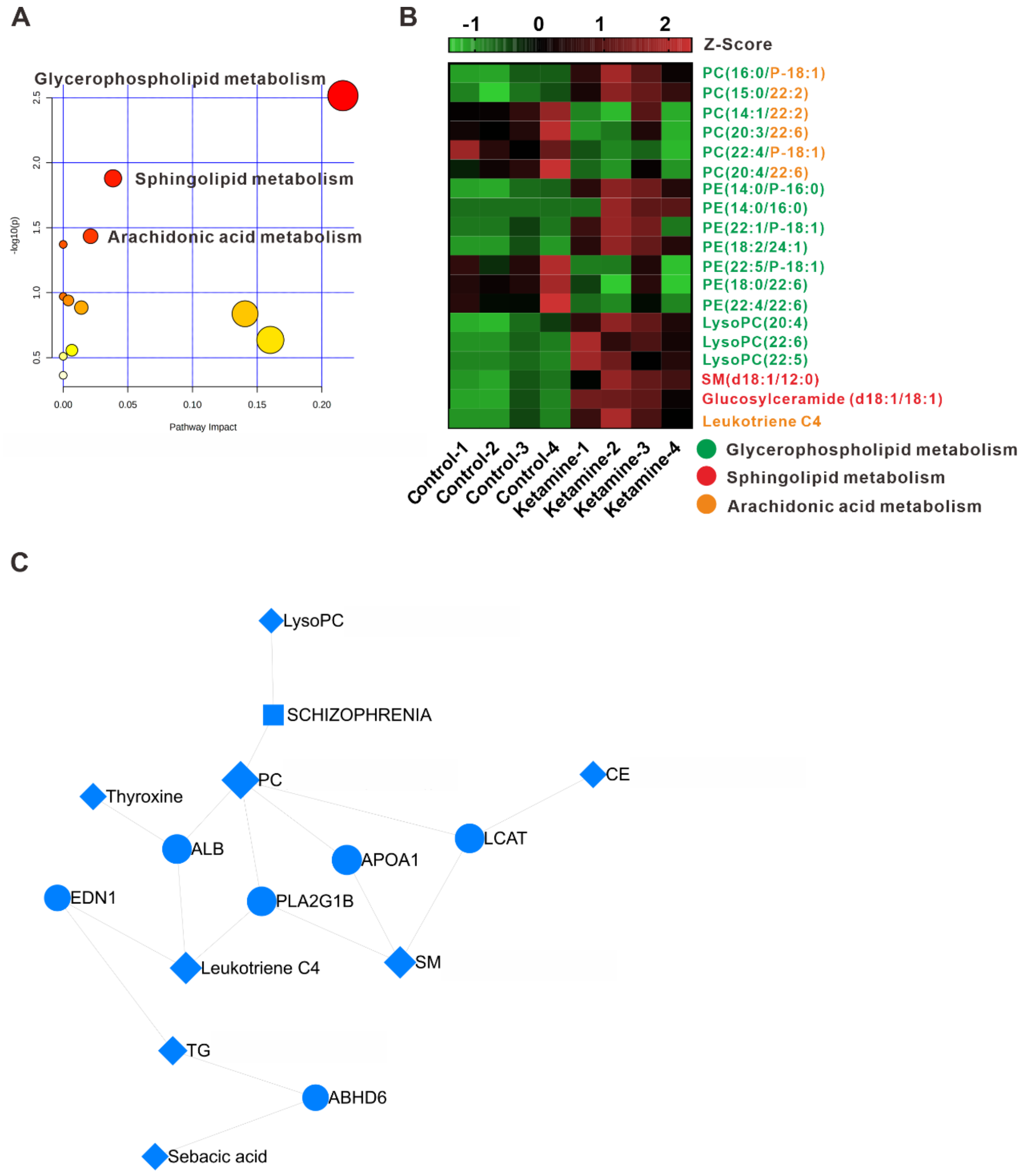

Figure S5. *(A) Pathway analysis using metaboanalyst5.0 based on KEGG. (B) Differentially expressed metabolites matching the Glycerophospholipid metabolism, Sphingolipid metabolism, and Linoleic acid metabolism. The data undergoes Z-Score conversion for more intuitively see the changes after s-ketamine injection, different colors represent the metabolic pathways to which they belong, the metabolites which have two color represents it belongs to not only one metabolic pathway (C). Metabolite-Gene-Disease Interaction Network based on metaboanalyst5.0.*

Table S1 *Differentially expressed metabolites detect based on KEGG data base.*

m/z	Ion type	Compound ID	Metabolites	ppm	P-value	Fold change	KEGG	Subregion
303.23	neg	HMDB0000207	Oleic acid	0.00	0.01	0.79	C00712	CE
327.23	neg	HMDB0002183	Docosahexaenoic acid	0.00	0.02	0.77	C06429	CE
331.26	neg	HMDB0002226	Adrenic acid	0.00	0.05	0.80	C16527	CE
347.04	neg	HMDB0001439	Phosphoribosyl formamidocarboxamide	0.85	0.02	0.64	C04734	CE
501.28	neg	HMDB0000653	Cholesterol sulfate	1.52	0.02	0.30	C18043	CE
580.43	pos	HMDB0010398	LysoPC(22:0)	4.34	0.05	2.14	C04230	CE
655.58	pos	HMDB0006726	CE(20:4)	4.54	0.02	2.27	C02530	CE
684.53	neg	HMDB0007961	PC(15:0/P-16:0)	0.00	0.02	2.55	C00157	CE
706.54	pos	HMDB0007869	PC(14:0/16:0)	0.31	0.04	2.24	C00157	CE
744.49	pos	HMDB0009215	PE(18:4/P-18:1)	2.60	0.01	0.28	C00350	CE
746.51	neg	HMDB0009116	PE(18:2/P-18:1)	0.00	0.03	0.77	C00350	CE
756.55	pos	HMDB0007881	PC(14:0/20:3)	0.00	0.03	0.65	C00157	CE
760.51	neg	HMDB0008835	PE(14:0/20:2)	0.00	0.04	0.79	C00350	CE
760.58	pos	HMDB0007879	PC(14:0/20:1)	2.48	0.03	0.67	C00157	CE
774.54	neg	HMDB0009644	PE(22:5/P-18:1)	0.00	0.05	0.82	C00350	CE
786.54	neg	HMDB0004866	Lactosylceramide (d18:1/12:0)	0.00	0.02	0.83	C01290	CE
806.57	pos	HMDB0007921	PC(14:1/22:2)	0.00	0.03	0.58	C00157	CE
830.51	pos	HMDB0009012	PE(18:0/22:6)	0.99	0.03	0.59	C00350	CE
832.51	neg	HMDB0009111	PE(18:2/22:6)	2.43	0.00	0.72	C00350	CE
835.53	neg	HMDB0009782	PI(16:0/18:1)	0.00	0.01	0.81	C00626	CE
835.60	pos	HMDB0009276	PE(20:1/22:6)	0.55	0.01	0.47	C00350	CE
857.52	neg	HMDB0009789	PI(16:0/20:4)	0.00	0.01	0.81	C00626	CE
857.58	pos	HMDB0009605	PE(22:4/22:6)	3.18	0.03	0.64	C00350	CE
859.53	neg	HMDB0009787	PI(16:0/20:3)	2.83	0.03	0.71	C00626	CE
862.65	neg	HMDB0007893	PC(14:0/24:0)	0.00	0.05	1.59	C00157	CE
872.56	pos	HMDB0008057	PC(18:0/22:6)	0.00	0.03	0.64	C00157	CE
883.53	neg	HMDB0009786	PI(16:0/20:2)	0.00	0.02	0.83	C00626	CE
916.53	pos	HMDB0008748	PC(22:6/22:6)	0.39	0.01	0.50	C00157	CE
917.52	pos	HMDB0009924	PIP(16:0/18:1)	3.41	0.00	0.41	C00626	CE
465.34	neg	HMDB0002972	Vitamin K1 2,3-epoxide	0.00	0.03	0.14	C05849	CTX
728.60	pos	HMDB0008060	PC(18:0/P-16:0)	2.46	0.05	1.63	C00157	CTX
728.60	pos	HMDB0008060	PC(18:0/P-16:0)	2.46	0.05	1.63	C00157	CTX
790.51	pos	HMDB0005779	PE(O-18:1/20:4)	1.63	0.01	0.64	C00350	CTX
131.03	neg	HMDB0000098	D-Xylose	3.74	0.05	0.72	C00181	HB
303.23	neg	HMDB0000207	Oleic acid	0.00	0.03	0.77	C00712	HB
834.62	neg	HMDB0007886	PC(14:0/22:0)	0.00	0.03	1.58	C00157	HB
834.62	neg	HMDB0008914	PE(15:0/24:0)	0.00	0.03	1.58	C00350	HB
844.64	neg	HMDB0008293	PC(20:0/P-18:1)	0.00	0.04	1.51	C00157	HB

845.64	neg	HMDB0013466	SM(d18:0/22:1)	0.00	0.04	1.64		C00550	HB
846.64	neg	HMDB0008191	PC(18:3/24:1)	4.47	0.05	1.70		C00157	HB
860.63	neg	HMDB0008588	PC(22:1/P-18:1)	0.00	0.02	1.71		C00157	HB
860.64	neg	HMDB0007894	PC(14:0/24:1)	0.00	0.04	1.75		C00157	HB
913.58	neg	HMDB0009817	PI(18:0/22:4)	4.39	0.02	1.44		C00626	HB
544.34	pos	HMDB0002815	LysoPC(18:1)	2.82	0.02	0.42		C04230	HP
699.50	neg	HMDB0007861	PA(18:0/18:2)	0.00	0.02	0.46		C00416	HP
750.54	pos	HMDB0009413	PE(20:4/P-18:1)	1.50	0.02	0.44		C00350	HP
760.58	pos	HMDB0007879	PC(14:0/20:1)	2.48	0.02	0.80		C00157	HP
776.56	pos	HMDB0009010	PE(18:0/22:5)	0.59	0.00	0.19		C00350	HP
783.56	pos	HMDB0008944	PE(16:0/22:5)	0.00	0.05	0.79		C00350	HP
860.63	neg	HMDB0008588	PC(22:1/P-18:1)	0.00	0.03	0.42		C00157	HP
100.11	pos	HMDB0031404	Cyclohexylamine	1.54	0.02	1.80		C00571	HYP
113.06	pos	HMDB0001164	trans-1,2-Dihydrobenzene-1,2-diol	0.14	0.02	2.17		C04221	HYP
117.09	pos	HMDB0000535	Caproic acid	0.43	0.04	3.14		C01585	HYP
671.47	neg	HMDB0007860	PA(16:0/18:2)	1.15	0.03	3.32		C00416	HYP
699.50	neg	HMDB0007861	PA(18:0/18:2)	0.00	0.04	2.18		C00416	HYP
714.54	pos	HMDB0007930	PC(14:1/P-18:1)	3.19	0.02	2.89		C00157	HYP
754.58	neg	HMDB0009281	PE(20:1/P-18:1)	0.00	0.03	2.48		C00350	HYP
766.61	pos	HMDB0009545	PE(22:1/P-18:1)	1.35	0.04	5.65		C00350	HYP
784.62	pos	HMDB0007952	PC(15:0/22:1)	1.98	0.02	2.31		C00157	HYP
788.54	neg	HMDB0008843	PE(14:0/22:2)	0.00	0.03	1.27		C00350	HYP
790.54	pos	HMDB0009045	PE(18:1/22:6)	0.28	0.03	0.61		C00350	HYP
819.69	pos	HMDB0008554	PC(22:0/P-16:0)	0.80	0.05	5.17		C00157	HYP
844.64	neg	HMDB0008293	PC(20:0/P-18:1)	0.00	0.02	3.08		C00157	HYP
846.64	neg	HMDB0008191	PC(18:3/24:1)	4.47	0.01	3.57		C00157	HYP
846.66	neg	HMDB0008292	PC(20:0/P-18:0)	0.00	0.03	4.05		C00157	HYP
860.58	neg	HMDB0008753	PC(22:6/P-18:1)	0.00	0.03	2.14		C00157	HYP
860.59	neg	HMDB0009113	PE(18:2/24:1)	0.00	0.01	2.28		C00350	HYP
860.63	neg	HMDB0008588	PC(22:1/P-18:1)	0.00	0.02	3.51		C00157	HYP
864.61	neg	HMDB0008654	PC(22:4/P-18:1)	0.00	0.04	2.95		C00157	HYP
888.66	neg	HMDB0008819	PC(24:1/P-18:1)	0.00	0.02	2.55		C00157	HYP
911.56	neg	HMDB0009791	PI(16:0/22:2)	0.00	0.05	1.67		C00626	HYP
181.08	pos	HMDB0000802	Pterin	0.36	0.05	0.62		C00715	MB
756.59	neg	HMDB0009248	PE(20:0/P-18:1)	0.00	0.04	1.41		C00350	MB
815.57	neg	HMDB0060370	3-demethylubiquinone-9	0.00	0.03	1.70		C03226	MB
842.59	neg	HMDB0009041	PE(18:1/22:2)	1.18	0.03	1.77		C00350	MB
874.72	pos	HMDB0008058	PC(18:0/24:0)	1.41	0.05	2.72		C00157	MB
329.27	neg	HMDB0000827	Stearic acid	1.95	0.01	0.55		C01530	OLF
501.28	neg	HMDB0000653	Cholesterol sulfate	1.52	0.04	0.50		C18043	OLF
767.54	pos	HMDB0012100	SM(d18:1/18:1)	3.19	0.03	0.32		C00550	OLF
790.51	pos	HMDB0005779	PE(O-18:1/20:4)	1.63	0.03	0.45		C00350	OLF

794.55	neg	HMDB0007879	PC(14:0/20:1)	0.00	0.04	2.06	C00157	OLF
834.54	pos	HMDB0007955	PC(15:0/22:4)	2.55	0.02	0.48	C00157	OLF
287.22	pos	HMDB0000672	Hexadecanedioic acid	3.66	0.01	6.82	C19615	PAL
302.06	neg	HMDB0000830	Neuraminic acid	0.00	0.01	1.74	C06469	PAL
367.36	neg	HMDB0002003	Tetracosanoic acid	1.36	0.04	0.20	C08320	PAL
399.27	neg	HMDB0010331	Palmitoyl glucuronide	1.03	0.03	46.32	C03033	PAL
553.44	pos	HMDB0002268	Alpha-Cryptoxanthin	1.63	0.01	2.71	C15981	PAL
567.30	pos	HMDB0030461	Hordatine A	2.54	0.05	4.29	C08307	PAL
582.30	pos	HMDB0010395	LysoPC(20:4)	2.88	0.00	1.97	C04230	PAL
602.30	pos	HMDB0000054	Bilirubin	0.00	0.00	3.37	C00486	PAL
603.30	pos	HMDB0030459	Hordatine B	0.00	0.01	2.43	C08308	PAL
643.34	pos	HMDB0001198	Leukotriene C4	0.00	0.03	4.26	C02166	PAL
643.34	pos	HMDB0005095	11-trans-Leukotriene C4	0.00	0.03	4.26	C02166	PAL
648.50	neg	HMDB0008850	PE(14:0/P-16:0)	2.23	0.03	2.62	C00350	PAL
655.14	pos	HMDB0003178	Heme	0.00	0.03	3.30	C00032	PAL
663.55	pos	HMDB0000885	CE(16:0)	1.55	0.01	4.04	C11251	PAL
664.54	pos	HMDB0012096	SM(d18:1/12:0)	0.00	0.02	2.64	C00550	PAL
671.47	neg	HMDB0007860	PA(16:0/18:2)	1.15	0.04	5.31	C00416	PAL
676.53	neg	HMDB0008851	PE(14:0/P-18:0)	0.00	0.02	2.38	C00350	PAL
678.35	pos	HMDB0001261	Coproporphyrinogen III	0.31	0.01	2.38	C03263	PAL
692.53	neg	HMDB0008826	PE(14:0/18:0)	4.52	0.01	4.47	C00350	PAL
693.34	pos	HMDB0012187	All-trans-heptaprenyl diphosphate	0.00	0.02	2.63	C04216	PAL
707.53	neg	HMDB0007901	PC(14:1/15:0)	0.40	0.01	4.31	C00157	PAL
708.58	pos	HMDB0004970	Glucosylceramide (d18:1/18:1)	0.00	0.01	2.65	C01190	PAL
714.54	pos	HMDB0007930	PC(14:1/P-18:1)	3.19	0.00	5.00	C00157	PAL
726.58	pos	HMDB0007996	PC(16:0/P-18:1)	0.66	0.00	3.14	C00157	PAL
727.18	pos	HMDB0029406	Amaranthin	2.43	0.00	2.93	C08537	PAL
728.60	pos	HMDB0008060	PC(18:0/P-16:0)	2.46	0.02	310.06	C00157	PAL
730.57	pos	HMDB0009017	PE(18:0/P-18:1)	3.05	0.00	2.23	C00350	PAL
746.51	pos	HMDB0009116	PE(18:2/P-18:1)	0.00	0.03	1.20	C00350	PAL
752.60	pos	HMDB0008095	PC(18:1/P-18:1)	1.28	0.00	3.19	C00157	PAL
756.59	pos	HMDB0009281	PE(20:1/P-18:1)	2.31	0.02	1.92	C00350	PAL
758.60	pos	HMDB0009248	PE(20:0/P-18:1)	1.68	0.03	3.65	C00350	PAL
759.64	pos	HMDB0012102	SM(d18:1/20:0)	2.24	0.00	2.42	C00550	PAL
766.65	pos	HMDB0004974	Glucosylceramide (d18:1/22:0)	4.38	0.02	3.06	C01190	PAL
770.65	pos	HMDB0009741	PE(24:0/P-16:0)	3.77	0.02	2.09	C00350	PAL
772.53	pos	HMDB0009012	PE(18:0/22:6)	1.57	0.01	1.21	C00350	PAL
778.62	pos	HMDB0010710	Galactosylceramide (d18:1/20:0)	3.09	0.01	3.66	C02686	PAL
796.62	pos	HMDB0008291	PC(20:0/P-16:0)	0.84	0.00	3.59	C00157	PAL
799.67	pos	HMDB0000248	Thyroxine	2.43	0.01	3.28	C01829	PAL

810.68	pos	HMDB0010712	Galactosylceramide (d18:1/24:1)	3.63	0.01	3.14	C02686	PAL
832.51	pos	HMDB0009111	PE(18:2/22:6)	2.43	0.01	1.38	C00350	PAL
836.69	pos	HMDB0008819	PC(24:1/P-18:1)	3.73	0.01	2.06	C00157	PAL
844.61	pos	HMDB0007954	PC(15:0/22:2)	2.20	0.04	0.54	C00157	PAL
847.69	pos	HMDB0007960	PC(15:0/24:1)	1.54	0.01	2.19	C00157	PAL
858.58	pos	HMDB0008654	PC(22:4/P-18:1)	4.80	0.01	0.37	C00157	PAL
859.53	neg	HMDB0009787	PI(16:0/20:3)	2.83	0.03	1.29	C00626	PAL
865.72	pos	HMDB0011698	SM(d18:1/26:0)	2.58	0.03	2.40	C00550	PAL
869.21	pos	HMDB0001041	2-Methylbutyryl-CoA	0.00	0.00	2.17	C15980	PAL
869.46	pos	HMDB0009939	PIP(16:1/16:1)	0.00	0.00	2.30	C00626	PAL
870.62	neg	HMDB0012318	3-O-Sulfogalactosylceramide (d18:1/24:1)	3.44	0.00	0.16	C06125	PAL
870.63	neg	HMDB0004834	Galabiosylceramide (d18:1/18:0)	0.64	0.02	0.19	C06126	PAL
924.74	pos	HMDB0008289	PC(20:0/24:0)	2.04	0.01	2.16	C00157	PAL
968.77	pos	HMDB0005478	TG(20:4/20:4/20:4)	1.56	0.00	3.16	C00422	PAL
90.09	pos	HMDB0032231	Dimethylethanolamine	2.20	0.03	0.32	C04308	STA
165.02	neg	HMDB0001866	3,4-Dihydroxymandelic acid	2.54	0.03	68.58	C05580	STA
181.08	pos	HMDB0000802	Pterin	0.36	0.04	0.58	C00715	STA
215.03	neg	HMDB0000122	D-Glucose	0.00	0.01	0.81	C00221	STA
258.28	pos	HMDB0001551	Palmitaldehyde	0.00	0.04	0.00	C00517	STA
790.54	pos	HMDB0009045	PE(18:1/22:6)	0.28	0.01	0.64	C00350	STA
796.62	pos	HMDB0008291	PC(20:0/P-16:0)	0.84	0.01	1.95	C00157	STA
848.64	neg	HMDB0007953	PC(15:0/22:0)	4.76	0.02	2.74	C00157	STA
143.11	pos	HMDB0013897	4-ene-Valproic acid	1.63	0.02	0.26	C16648	TH
195.05	neg	HMDB0000098	D-Xylose	4.86	0.01	2.27	C00181	TH
846.66	neg	HMDB0008292	PC(20:0/P-18:0)	0.00	0.03	1.64	C00157	TH

Table S2 *Differentially expressed metabolites detect in ventral pallidal.*

m/z	Ion mode	Compound ID	Metabolites	ppm	P-value	Fold change	KEGG
185.12	neg	HMDB0033217	(2xi,6xi)-7-Methyl-3-methylene-1,2,6,7-octanetetrol	2.17	0.03	0.31	NA
219.98	neg	HMDB0000272	Phosphoserine	4.34	0.01	6.00	C01005
365.16	pos	HMDB0060494	N-Acetylmuramoyl-Ala	0.00	0.01	5.74	C02999
532.28	pos	HMDB0010383	LysoPC(16:1)	0.68	0.02	11.32	C04230
542.26	pos	HMDB0011484	LPE(20:3)	0.44	0.03	16.25	NA
544.28	pos	HMDB0011483	LPE(20:2)	0.71	0.03	17.23	NA
550.28	pos	HMDB0241887	(5E)-7-{4,6-Dihydroxy-2-[(1E,5E)-3-hydroxyocta-1,5-dien-1-yl]oxan-3-yl}hept-5-enoylcarnitine	3.35	0.05	9.63	NA
551.97	pos	HMDB0006821	2,5-Diaminopyrimidine nucleoside triphosphate	1.56	0.04	16.34	C05923
553.44	pos	HMDB0002268	Alpha-Cryptoxanthin	1.63	0.04	8.22	C15981
568.28	pos	HMDB0011493	LPE(22:4)	3.07	0.03	17.30	NA
569.96	pos	HMDB0001364	Adenosine tetraphosphate	4.22	0.02	130.86	C03483
580.43	pos	HMDB0010398	LysoPC(22:0)	4.34	0.04	7.96	C04230
587.01	pos	HMDB0012300	UDP-4-dehydro-6-deoxy-D-glucose	1.70	0.01	4.95	C04089
590.32	pos	HMDB0010404	LysoPC(22:6)	0.87	0.05	11.80	C04230
602.30	pos	HMDB0000054	Bilirubin	0.00	0.04	7.25	C00486
602.48	pos	HMDB0007056	DG(34:6)	0.06	0.04	18.31	NA
604.47	pos	HMDB0040132	N-(2R-Hydroxyhexadecanoyl)-2S-amino-9-methyl-4E,8E-octadecadiene-1,3R-diol	0.73	0.02	17.86	NA
607.47	pos	HMDB0007013	DG(32:0)	4.75	0.02	5.69	NA
609.45	pos	HMDB0007338	DG(36:8)	3.93	0.05	31.57	NA
632.18	pos	HMDB0001176	Cytidine monophosphate N-acetylneuraminic acid	0.82	0.03	16.61	C00128
636.32	pos	HMDB0256524	PI(20:5)	1.10	0.01	5.14	NA
637.48	pos	HMDB0007288	DG(38:8)	0.65	0.05	9.81	NA
638.33	pos	HMDB0061690	LysoPI(20:4)	0.00	0.04	8.05	NA
641.32	pos	HMDB0012993	Leukotriene C5	0.00	0.02	7.30	NA
641.51	pos	HMDB0007121	DG(38:6)	0.00	0.03	7.90	NA
649.58	pos	HMDB0007065	DG(38:2)	0.43	0.01	29.94	NA
655.14	pos	HMDB0003178	Heme	0.00	0.05	57.61	C00032
659.33	pos	HMDB0013058	S-(9-deoxy-delta9,12-PGD2)-glutathione	0.00	0.03	9.25	NA

664.54	pos	HMDB0012096	SM(d18:1/12:0)	0.00	0.04	21.70	C00550
673.35	pos	HMDB0116536	PGP(24:0)	0.00	0.03	9.86	NA
676.53	pos	HMDB0008851	PE(14:0/P-18:0)	0.00	0.01	17.31	C00350
678.35	pos	HMDB0001261	Coproporphyrinogen III	0.31	0.02	13.16	C03263
685.55	pos	HMDB0061670	CE(DiMe(9,3))	0.00	0.02	7.84	NA
692.53	pos	HMDB0008826	PE(14:0/18:0)	4.52	0.03	14.33	C00350
693.34	pos	HMDB0012187	All-trans-heptaprenyl diphosphate	0.00	0.03	35.88	C04216
694.15	pos	HMDB0001312	Diadenosine diphosphate	0.00	0.01	8.78	NA
700.53	pos	HMDB0112953	PE-NMe(33:1)	0.11	0.04	8.43	NA
707.53	pos	HMDB0007901	PC(14:1/15:0)	0.40	0.04	62.98	C00157
708.58	pos	HMDB0004970	Glucosylceramide (d18:1/9Z-18:1)	0.00	0.03	10.48	C01190
711.59	pos	HMDB0116449	DG(41:3)	4.01	0.02	37.56	NA
714.54	pos	HMDB0007930	PC(14:1/P-18:1)	3.19	0.02	39.11	C00157
720.55	pos	HMDB0007935	PC(15:0/16:0)	0.00	0.04	11.89	C00157
726.58	pos	HMDB0007996	PC(16:0/P-18:1)	0.66	0.03	11.91	C00157
729.57	pos	HMDB0116370	DG(11D14D14)	0.00	0.03	8.11	NA
729.58	pos	HMDB0001060	Ubiquinol 8	0.00	0.04	9.06	NA
729.64	pos	HMDB0042279	TG(44:2)	3.89	0.04	12.38	NA
730.21	pos	HMDB0000445	Alpha-Tetrasaccharide	4.62	0.05	29.33	C06768
730.57	pos	HMDB0009017	PE(18:0/P-18:1)	3.05	0.05	6.24	C00350
738.19	pos	HMDB0001902	Pteroyltriglutamic acid	0.00	0.02	9.59	NA
749.49	neg	HMDB0114826	Phosphatidate(37:2)	1.29	0.00	2.62	NA
751.50	neg	HMDB0114825	PA(37:1)	1.33	0.05	3.45	NA
752.60	pos	HMDB0008095	PC(18:1/P-18:1)	1.28	0.05	9.10	C00157
756.59	pos	HMDB0009281	PE(20:1/P-18:1)	2.31	0.02	8.23	C00350
758.60	pos	HMDB0009248	PE(20:0/P-18:1)	1.68	0.04	24.88	C00350
759.64	pos	HMDB0012102	SM(d18:1/20:0)	2.24	0.03	12.49	C00550
762.53	neg	HMDB0012356	PS(34:0)	0.00	0.04	1.27	NA
764.60	pos	HMDB0009578	PE(22:2/P-18:1)	0.00	0.04	16.10	C00350
770.65	pos	HMDB0009741	PE(24:0/P-16:0)	3.77	0.02	11.80	C00350
774.60	pos	HMDB0007945	PC(15:0/20:1)	2.24	0.03	6.15	C00157
776.56	pos	HMDB0009010	PE(18:0/22:5)	0.59	0.00	0.14	C00350
779.41	pos	HMDB0115051	Phosphatidate(40:10)	2.37	0.03	18.90	NA
782.61	pos	HMDB0007954	PC(15:0/22:2)	0.33	0.03	6.92	C00157
792.42	pos	HMDB0012350	PS(34:5)	0.00	0.02	8.46	NA
799.67	pos	HMDB0000248	Thyroxine	2.43	0.04	30.45	C01829
800.61	pos	HMDB0114972	Phosphatidate(42:3)	4.13	0.01	14.55	NA
810.68	pos	HMDB0010712	Galactosylceramide (d18:1/24:1(15Z))	3.63	0.03	47.29	C02686
814.55	neg	HMDB0009610	PE(22:4/P-18:0)	0.00	0.00	0.22	C00350
814.63	pos	HMDB0007927	PC(14:1/24:1)	3.19	0.04	7.03	C00157

825.68	pos	HMDB0115268	PA(46:1)	3.72	0.01	9.73	NA
836.69	pos	HMDB0008819	PC(24:1/P-18:1)	3.73	0.01	7.13	C00157
847.45	pos	HMDB0013478	PGP(34:3)	0.00	0.03	9.06	NA
847.69	pos	HMDB0007960	PC(15:0/24:1)	1.54	0.02	7.63	C00157
858.60	pos	HMDB0113278	MMPE(42:5)	2.10	0.03	0.08	NA
858.70	pos	HMDB0114010	DMPE(42:1)	4.87	0.01	10.72	NA
859.53	neg	HMDB0009787	PI(16:0/20:3)	2.83	0.03	1.40	C00626
865.50	neg	HMDB0116602	PG(42:9)	0.00	0.05	1.26	NA
865.72	pos	HMDB0011698	SM(d18:1/26:0)	2.58	0.03	56.73	C00550
866.51	neg	HMDB0061552	PS(DiMe(11,3)/DiMe(11,3))	4.68	0.02	1.36	NA
869.21	pos	HMDB0001041	2-Methylbutyryl-CoA	0.00	0.01	8.58	C15980
869.46	pos	HMDB0009939	PIP(16:1/16:1)	0.00	0.02	13.31	C00626
869.70	pos	HMDB0043451	TG(55:9)	0.95	0.01	10.01	NA
870.21	pos	HMDB0002185	3-trans,5-cis-Octadienoyl-CoA	0.48	0.02	21.82	NA
880.45	pos	HMDB0116064	CDDG(P-28:0)	0.00	0.01	10.33	NA
887.47	pos	HMDB0116797	CL(32:0)	4.12	0.02	60.25	NA
913.73	pos	HMDB0043538	TG(57:10)	0.80	0.02	9.20	NA
924.74	pos	HMDB0008289	PC(20:0/24:0)	2.04	0.02	8.61	C00157
925.82	pos	HMDB0043096	TG(57:4)	0.78	0.03	19.55	NA
935.74	pos	HMDB0043067	TG(55:4)	3.41	0.02	16.34	NA
942.76	pos	HMDB0010510	TG(58:11)	4.59	0.01	30.03	NA
968.54	pos	HMDB0116076	CDDG(P-33:0)	0.00	0.04	14.32	NA
968.77	pos	HMDB0005478	TG(20:4)	1.56	0.02	43.68	C00422
969.51	pos	HMDB0116799	CL(35:0)	0.00	0.03	10.39	NA
984.51	pos	HMDB0006973	CDDG(P-36:4)	0.00	0.03	26.37	NA

Table S3 *Differentially expressed metabolites detect in medial pallidal.*

m/z	Ion mode	Compound ID	Metabolites	ppm	P-value	Fold change	KEGG
536.30	pos	HMDB0241583	(5Z,8Z,13E,15S)-11,12,15-Trihydroxyicosa-5,8,13-trienoylcarnitine	2.81	0.04	0.03	NA
545.26	pos	HMDB0034229	Hygromycin B	3.88	0.05	0.07	C01925
549.29	pos	HMDB0002379	Mesoporphyrin IX	2.56	0.04	0.14	NA
558.28	pos	HMDB0241614	(4Z,7R,8E,10Z,12E,14E,17S,19Z)-7,16,17-Trihydroxydocosa-4,8,10,12,14,19-hexaenoylcarnitine	0.02	0.02	0.10	NA
565.48	pos	HMDB0007016	DG(32:2)	0.77	0.04	0.07	NA
568.45	pos	HMDB0002890	3-cis-Hydroxy-b,e-Caroten-3'-one	1.46	0.03	0.13	NA
569.16	pos	HMDB0304224	6-(alpha-D-glucosaminyl)-1-phosphatidyl-1D-myo-inositol	1.19	0.05	0.03	NA
569.96	pos	HMDB0001364	Adenosine tetraphosphate	4.22	0.03	0.06	C03483
578.48	pos	HMDB0007019	DG(32:4)	2.61	0.04	0.06	NA
579.50	pos	HMDB0007074	DG(33:2)	0.96	0.03	0.03	NA
582.30	pos	HMDB0010395	LysoPC(20:4)	2.88	0.05	0.26	C04230
587.47	pos	HMDB0007027	DG(34:5)	0.87	0.04	0.14	NA
590.32	pos	HMDB0010404	LysoPC(22:6)	0.87	0.03	0.07	C04230
590.49	pos	HMDB0240678	Cer(d35:1)	1.69	0.04	0.04	NA
591.31	pos	HMDB0002430	(3a,5b,7a)-23-Carboxy-7-hydroxy-24-norcholan-3-yl-b-D-Glucopyranosiduronic acid	3.90	0.03	0.10	NA
592.34	pos	HMDB0010402	LysoPC(22:5)	2.44	0.04	0.04	C04230
598.16	pos	HMDB0060391	4,5-Dihydro-4-hydroxy-5-S-glutathionyl-benzo[a]pyrene	4.98	0.05	0.02	C14855
604.47	pos	HMDB0040132	N-(2R-Hydroxyhexadecanoyl)-2S-amino-9-methyl-4E,8E-octadecadiene-1,3R-diol	0.73	0.04	0.14	NA
604.53	pos	HMDB0241645	Nonacosanoylcarnitine	1.91	0.02	0.04	NA
621.49	pos	HMDB0007150	DG(38:7)	2.23	0.04	0.12	NA
627.50	pos	HMDB0007092	DG(37:6)	1.94	0.04	0.01	NA
631.55	pos	HMDB0112216	CE(5M7)	0.25	0.04	0.03	NA
632.18	pos	HMDB0001176	Cytidine monophosphate N-acetylneuraminic acid	0.82	0.05	0.13	C00128
635.56	pos	HMDB0064779	TG(37:0)	3.52	0.04	0.02	NA
637.48	pos	HMDB0007288	DG(38:8)	0.65	0.01	0.03	NA
643.34	pos	HMDB0001198	Leukotriene C4	0.00	0.05	0.10	C02166
643.38	pos	HMDB0002579	Glycochenodeoxycholic acid 3-glucuronide	3.59	0.04	0.01	C03033

648.50	pos	HMDB0008850	PE(14:0/P-16:0)	2.23	0.04	0.12	C00350
649.35	pos	HMDB0116660	PG(24:0)	0.60	0.04	0.06	NA
655.14	pos	HMDB0003178	Heme	0.00	0.05	0.08	C00032
655.58	pos	HMDB0006726	CE(20:4)	4.54	0.04	0.00	C02530
656.34	pos	HMDB0013022	Neuromedin N	0.00	0.03	0.15	NA
657.52	pos	HMDB0112214	CE(5D5)	3.59	0.05	0.07	NA
663.55	pos	HMDB0000885	CE(16:0)	1.55	0.04	0.03	C11251
664.54	pos	HMDB0012096	SM(d18:1/12:0)	0.00	0.04	0.12	C00550
669.56	pos	HMDB0010369	CE(18:3)	0.00	0.04	0.05	NA
673.35	pos	HMDB0116536	PGP(24:0)	0.00	0.05	0.16	NA
681.52	pos	HMDB0008824	PE(14:0/16:0)	1.51	0.01	0.03	C00350
682.52	pos	HMDB0031983	Lucyobroside	3.67	0.04	0.14	NA
685.55	pos	HMDB0061670	CE(DiMe(9,3))	0.00	0.04	0.17	NA
695.57	pos	HMDB0006726	CE(20:4(5Z,8Z,11Z,14Z))	0.67	0.03	0.06	C02530
699.57	pos	HMDB0061672	CE(monome)	2.24	0.03	0.10	NA
699.61	pos	HMDB0010374	CE(22:5)	1.51	0.04	0.02	NA
702.58	pos	HMDB0010703	CerP(d40:1)	4.65	0.03	0.11	NA
708.58	pos	HMDB0004970	Glucosylceramide (d18:1/9Z-18:1)	0.00	0.04	0.11	C01190
714.37	pos	HMDB0012342	PS(28:2)	0.00	0.03	0.11	NA
726.58	pos	HMDB0007996	PC(16:0/P-18:1)	0.66	0.05	0.14	C00157
734.57	pos	HMDB0114825	PA(37:1)	4.23	0.03	1.34	NA
738.19	pos	HMDB0001902	Pteroyltriglutamic acid	0.00	0.03	0.15	NA
753.39	pos	HMDB0115044	Phosphatidate(38:9)	4.63	0.04	0.13	NA
755.40	pos	HMDB0114990	PA(38:8)	0.00	0.04	0.14	NA
766.61	pos	HMDB0009545	PE(22:1/P-18:1)	1.35	0.04	0.08	C00350
779.41	pos	HMDB0115051	Phosphatidate(40:10)	2.37	0.04	0.11	NA
782.61	pos	HMDB0007954	PC(15:0/22:2)	0.33	0.05	0.22	C00157
799.67	pos	HMDB0000248	Thyroxine	2.43	0.05	0.14	C01829
806.57	pos	HMDB0007921	PC(14:1/22:2)	0.00	0.05	1.55	C00157
808.62	pos	HMDB0009113	PE(18:2/24:1)	0.28	0.03	0.07	C00350
814.51	pos	HMDB0009644	PE(22:5/P-18:1)	4.93	0.04	2.18	C00350
825.54	pos	HMDB0115293	PA(44:7)	0.00	0.01	1.75	NA
830.51	pos	HMDB0009012	PE(18:0/22:6)	0.99	0.05	1.57	C00350
856.59	pos	HMDB0008387	PC(20:3/22:6)	0.00	0.03	1.99	C00157
857.58	pos	HMDB0009605	PE(22:4/22:6)	3.18	0.01	3.97	C00350
858.58	pos	HMDB0008654	PC(22:4/P-18:1)	4.80	0.00	4.81	C00157
858.60	pos	HMDB0113278	MMPE(42:5)	2.10	0.02	2.93	NA
863.72	pos	HMDB0113108	MMPE(42:0)	1.16	0.05	0.06	NA
887.47	pos	HMDB0116797	CL(32:0)	4.12	0.05	0.14	NA
892.52	pos	HMDB0008452	PC(20:4/22:6)	4.40	0.02	4.52	C00157
968.77	pos	HMDB0005478	TG(20:4/20:4/20:4)	1.56	0.04	0.17	C00422
